# Proteomics-Based Data Integration of Wheat Cultivars Facing *Fusarium graminearum* Strains Revealed a Core-Responsive Pattern Controlling Fusarium Head Blight

**DOI:** 10.3389/fpls.2021.644810

**Published:** 2021-05-31

**Authors:** Francis Fabre, Serge Urbach, Sylvie Roche, Thierry Langin, Ludovic Bonhomme

**Affiliations:** ^1^Université Clermont Auvergne, INRAE, UMR 1095 Génétique Diversité Ecophysiologie des Céréales, Clermont-Ferrand, France; ^2^Institut de Génomique Fonctionnelle, Université de Montpellier, CNRS, INSERM, Montpellier, France; ^3^INRAE, Unité Experimentale 1375, Phénotypage au Champ des Céréales (PHACC), Clermont-Ferrand, France

**Keywords:** *Triticum aestivum*, susceptibility factors, Fusarium head blight, strain aggressiveness, plant–pathogen interactions

## Abstract

Fusarium head blight (FHB), mainly occurring upon *Fusarium graminearum* infection in a wide variety of small-grain cereals, is supposed to be controlled by a range of processes diverted by the fungal pathogen, the so-called susceptibility factors. As a mean to provide relevant information about the molecular events involved in FHB susceptibility in bread wheat, we studied an extensive proteome of more than 7,900 identified wheat proteins in three cultivars of contrasting susceptibilities during their interaction with three *F. graminearum* strains of different aggressiveness. No cultivar-specific proteins discriminated the three wheat genotypes, demonstrating the establishment of a core proteome regardless of unequivocal FHB susceptibility differences. Quantitative protein analysis revealed that most of the FHB-induced molecular adjustments were shared by wheat cultivars and occurred independently of the *F. graminearum* strain aggressiveness. Although subtle abundance changes evidenced genotype-dependent responses to FHB, cultivar distinction was found to be mainly due to basal abundance differences, especially regarding the chloroplast functions. Integrating these data with previous proteome mapping of the three *F. graminearum* strains facing the three same wheat cultivars, we demonstrated strong correlations between the wheat protein abundance changes and the adjustments of fungal proteins supposed to interfere with host molecular functions. Together, these results provide a resourceful dataset that expands our understanding of the specific molecular events taking place during the wheat–*F. graminearum* interaction.

## Introduction

Fusarium head blight (FHB), firstly described by Smith (1884), is a microbial disease associated with several fungal species from the Fusarium and *Microdochium* genera that affect small-grain cereals throughout the world (Parry et al., 1995; Goswami and Kistler, 2004). In many wheat-growing regions such as the United States, Europe, China, and Canada, epidemics are dominated by the occurrence of *Fusarium graminearum* Schwabe (Hypocreales: Nectriaceae) (teleomorph: *Gibberella zeae*). Due to intensified outbreaks promoted upon the global climate change, FHB has become a major issue to sustain the ever-increasing human food needs (Zhang et al., 2012; AlTaweel et al., 2017; Timmusk et al., 2020). Global losses attributed to FHB on bread wheat were estimated at more than United States $250 billion per year between 2011 and 2014 (Wilson et al., 2017), in particular by affecting the nutritional quality of grains and causing major health risks through mycotoxin contamination of crops (Li et al., 2014).

In the past decades, substantial efforts have been devoted to the identification of resistance sources to FHB in wheat (Buerstmayr et al., 2020; Ma et al., 2020). Over the last 20 years, the mapping of several association panels has made possible the identification of more than 620 resistance quantitative trait loci (QTLs), delineating 77 meta-QTLs distributed over all the chromosomes of bread wheat (Steiner et al., 2017, 2019; Venske et al., 2019; Zheng et al., 2020). Although many determinants were already described as associated with wheat FHB resistance, the specific molecular mechanisms responsible for FHB resistance remain poorly understood (Wang et al., 2019), as exemplified by the QTL *Fhb1* characterized as the most stable and efficient locus for wheat resistance to FHB (Bai et al., 1999; Ollier et al., 2020). While recent studies have shown that different mutations inducing the *TaHRC* gene loss of function explain part of this *Fhb1*-mediated resistance (Li et al., 2019; Su et al., 2019), further works have either suggested a role for the *WFhb1-1* gene through its potential antifungal activity (Paudel et al., 2020) or for the *TaLAC4* gene whose mutation leads to an increase of FHB susceptibility (Soni et al., 2020). In addition to the current sources of FHB resistance, several works introduced alternative forms of resistance *via* the identification of wheat genes involved in the success of the disease (reviewed in Fabre et al., 2020). By definition, any plant gene that facilitates infection and that promotes compatibility with pathogens can be considered as a susceptibility gene (van Schie and Takken, 2014). Formally (Li et al., 2019; Su et al., 2019; Brauer et al., 2020; Su et al., 2021) or indirectly identified (Ma et al., 2006; Basnet et al., 2012; Garvin et al., 2015; Hales et al., 2020), the deletion of these genes coding for the so-called susceptibility factors may provide a complementary approach to the introgression of gain-of-function resistance genes (Fabre et al., 2020; Moniruzzaman et al., 2020; Gorash et al., 2021). However, despite these attempts to elucidate the molecular processes involved both in wheat resistance and susceptibility to FHB, understanding this interaction still requires many efforts, in particular by reconciling information from the two partners of this pathosystem.

In a previous work, a dual-proteomics analysis of the interaction between the aggressive *F. graminearum* strain MDC_Fg1 and the susceptible wheat cultivar Recital allowed the identification of extensive co-variations in the dynamics of both fungal and wheat protein abundances during the 48–72 h post-inoculation (hpi) transition phase (Fabre et al., 2019b). Along with the amplification of the plant’s stress responses, the wide remodeling of putative fungal effector synthesis during these particular time points depicted a specific molecular dialogue able to drive the fate of the infection process. Based on this primary result, an additional study was carried out at 72 hpi to characterize the proteome specificities of three *F. graminearum* strains facing three wheat cultivars respectively harboring contrasting aggressiveness and susceptibilities (Fabre et al., 2019a). In accordance with previous works that have observed strong links between genetic polymorphism and the aggressiveness of *F. graminearum* strains (Bai and Shaner, 1996; Carter et al., 2000; Xue et al., 2004; Goswami and Kistler, 2005; Malbrán et al., 2012; Talas et al., 2012; Garmendia et al., 2018; Shin et al., 2018), this study showed that the aggressiveness of the three fungal strains was closely related with their ability to produce putative effectors in large quantities without any major influence from the host genetics (Fabre et al., 2019a). On the plant side, although several transcriptomic studies have already demonstrated that different wheat cultivars of varying resistance levels exhibited differential responses to the disease (Bernardo et al., 2007; Erayman et al., 2015; Zega and D’Ovidio, 2016; Pan et al., 2018; Wang et al., 2018; Brauer et al., 2019b), only a few large-scale proteomic studies have already been carried out to understand the molecular specificities of wheat responses to FHB (Wang et al., 2019; Yang et al., 2021), and their relationships with strain aggressiveness have not yet been described.

Here, the aim of this work was to dissect the plant component of the dual proteome established during the FHB process using the same three wheat cultivars facing the three fungal strains as described in Fabre et al. (2019a). Qualitative and quantitative dissection of the three wheat cultivar proteomes was devoted to identify the molecular events that drive the FHB susceptibility differences. This included the identification of (i) the generic molecular adjustments taking place during FHB progress; (ii) the cultivar-specific responses and their accommodation with different *F. graminearum* strains inducing FHB; and (iii) the range of wheat proteins that basically discriminate the three wheat cultivars of contrasting susceptibilities. The joint analysis of all these data with the fungal protein information was carried out to identify the relationship between wheat protein abundance changes and fungal effectors differentially accumulated between the three *F. graminearum* strains (Fabre et al., 2019a).

## Materials and Methods

### Plant Growth and *F. graminearum* Inoculation

The three wheat cultivars (cv) used in this experiment were selected from previous field observations for their contrasting susceptibilities to FHB, including, in ascending order of susceptibility, cv. Renan, cv. Cadenza, and cv. Recital. Recital and Renan are known to be among the most contrasting cultivars of the French wheat collections for their responses to FHB, while Cadenza is considered as intermediate (Gervais et al., 2003; Zwart et al., 2008; Fabre et al., 2019a). For each wheat cultivar, the seeds were sown in buckets and kept at 20°C to allow germination. Plant vernalization was carried out at 4°C for 8 weeks, then they were transplanted in 4-L pots and transferred to a growth cabinet with optimal conditions to allow tillering and synchronized flowering. For each wheat cultivar, 12 plants were prepared and divided into three randomized complete blocks in the growth cabinet. Each block was surrounded by additional plants to control any edge effects. Automatic watering was installed, and the daily photoperiod was set at 16-h daylight for a temperature of 20°C and 8-h darkness at 18°C. Relative humidity was maintained at 80% during day and night. After 47 days, flowering of the main culm was observed in all plants. Seven additional days were awaited for *F. graminearum* infection in order to inoculate spikes showing the same ontogeny during the same day (mid-anthesis).

Three *F. graminearum* French strains, named MDC_Fg1, MDC_Fg13, and MDC_FgU1, were selected for their contrasting aggressiveness based on a previous study (Fabre et al., 2019a). In this previous work, fungal aggressiveness was characterized through the monitoring of symptom severity induced by each *F. graminearum* strain individually inoculated in the three wheat cultivars. This profiling allowed for the establishment of an unambiguous aggressiveness ranking, where the MDC_Fg1 strain produced systematically the strongest symptoms, MDC_Fg13 strain induced intermediate ones, and MDC_FgU01 strain produced the weakest ones (Fabre et al., 2019a). This ranking has further proven the strong relationships with fungal protein abundance differences and especially with the accumulation of effector proteins. For each *F. graminearum* strain, the inocula were prepared at a concentration of 10^5^ spores/ml of water. The three strains were individually inoculated in three plants of each wheat cultivar, i.e., three plants × three *F. graminearum* strains for a total of nine plants per cultivar. For each cultivar, inoculation was performed at the mid-anthesis stage by depositing 10 μl of inoculum in the floral cavity of six contiguous spikelets located in the middle zone of three synchronized spikes per plant, as described in Fabre et al. (2019b). Three other plants per cultivar were inoculated with water following the same methodology and were used as controls. For each cultivar × strain combination, the point-inoculated spikelets of the three spikes of three independent plants were specifically collected 72 hpi. The 72-hpi time point was chosen on the basis of our previous analyses that highlighted synchronized regulation in both fungal and wheat proteomes, demonstrating massive changes in protein abundance as compared to the 48-hpi stage (Fabre et al., 2019b). For each cultivar × strain combination and control sample, three biological replicates corresponding to three individual plants characterized by the pool of all inoculated spikelets from three spikes were collected and stored at −80°C for proteomics.

### Protein Extraction and Mass Spectrometry Analyses

For each biological replicate, wheat spikelets were finely ground in liquid nitrogen. Denaturing protein extraction was performed on 100 mg of ground material using 1.8 ml of cold trichloroacetic acid/β-mercaptoethanol/acetone solution and then mixed and stored at −20°C for 1 h, as described in Bonhomme et al. (2012). Protein resolubilization was performed in a urea–thiourea buffer [6 M urea, 2 M thiourea, 1% Halt Protease Inhibitor Cocktail 100X (78429; Thermo Fisher Scientific), 100 mM ammonium bicarbonate, and 0.1% ProteaseMAX Surfactant (V2071; Promega)] by following the ratio 10 μl/mg of dry matter, as described in Fabre et al. (2019b). Protein quantification was performed using the Protein Quantification Assay kit (740967.250; Macherey-Nagel) with bovine serum albumin as the standard. For each sample, a starting amount of 600 μg of proteins from each sample was collected and submitted to reduction and alkylation, as described in Maghames et al. (2018). Protein digestion was achieved using trypsin (Promega) following a protease/protein ratio of 1:30. Tandem mass spectrometry (MS/MS) analyses were performed using a nanoESI Q ExactiveTM HF-X Hybrid Quadrupole-Orbitrap^TM^ mass spectrometer (0726042; Thermo Fisher Scientific) coupled with an Ultimate 3000 HPLC (Thermo Fisher Scientific). The high-performance liquid chromatography (HPLC) gradients and data acquisition parameters were set as described in Fabre et al. (2019a).

### Identification and Quantification of Peptides and Proteins From MS/MS Data

Database searches were performed using X!Tandem (^1^ 2010.01.01). Enzymatic cleavage was described for trypsin digestion with one possible miscleavage. Cys-carboxyamidomethylation and Met oxidation were set as static and variable modifications, respectively. The precursor mass and fragment mass tolerance were 10 ppm and 0.5 Da, respectively. Protein identifications were performed using a concatenated file including the wheat database (International Wheat Genome Sequencing Consortium [IWGSC], 2018 v1.0, 110,790 entries^2^, April 2017) and a contaminant database (trypsin, keratins, etc.). To prevent peptides derived from *F. graminearum* proteins from being assigned to plant proteins, the MDC_Fg1 (13,166 entries, January 2019), MDC_Fg13 (13,297 entries, January 2019), and MDC_FgU1 (13,014 entries, January 2019) databases obtained from an in-lab resequencing of each *F. graminearum* strain (Alouane et al., 2018) were also added for the protein identifications. Identified proteins were parsed and grouped using the X!TandemPipeline v0.2.40 c + + (Langella et al., 2017). Data filtering was achieved according to a peptide *E* value < 0.05. Proteins were reported when they displayed at least two different peptides in the same sample and when the protein *E* value is < 0.0001. The false discovery rate (FDR) at the peptide level assessed from searches against reversed amino acid sequences for each protein was smaller than 0.8 × 10^–6^. Relative protein abundance was determined from the sum of the abundances of each specific peptide assigned to a given protein using MassChroQ 2.2.17 software (Valot et al., 2011) by extraction of the ion chromatograms as described in Bonhomme et al. (2012). Protein abundance normalization was then performed by dividing the ratios by the total peptide abundance value in each LC-MS/MS run. Subsequent statistical analyses were performed on log2-transformed normalized data.

### Statistical Analyses

Statistical analyses were performed using the programming software R 3.4.4 (R Core Team, 2018). Principal component analysis (PCA) using samples as individuals and based on all the quantification values for each protein was performed in order to identify the main factors explaining the differences between samples. PCA was computed using Z-score transformed values and established from the correlation matrix.

At the individual protein level, analysis of the explanatory factors for wheat protein abundance variations was carried out, after verification of the absence of any block/repeat effect, using a nested analysis of variance (ANOVA) test based on the following linear model:

$$Y_{i\,j\,k\,l}=\mu +C\,v_{i}+T_{j}+\left(C\,v_{i}\times T_{j}\right)+\left(C\,v_{i}\times T_{j}\,\left\{S_{k}\right\}\right)+\varepsilon_{i\,j\,k\,l}$$

where *Y*_*ijkl*_ refers to the individual values, μ is the general mean of the variable considered, *Cv*_*i*_ is the effect of the wheat cultivar (i.e., cv. Recital, cv. Cadenza, and cv. Renan), *T_j_* is the effect of the treatment (i.e., *F. graminearum*-inoculated or water-inoculated), *Cv*_*i*_ × *T_j_* is the interaction of the cultivar effect by the treatment, *Cv*_*i*_ × *T_j_* {*S*_*k*_} is the effect attributable to the interaction of the two main factors (*Cv*_*i*_ × *T_j_*) taking into account the inoculated fungal strain (*S*_*k*_) as a nested factor in the main treatment factor (*T_j_*), and ε_*ijkl*_ is the residual.

For each individual wheat protein, the *p* values obtained for each effect (*Cv*_*i*_, *T_j_*, *Cv*_*i*_ × *T_j_*, and *Cv*_*i*_ × *T_j_* {*S*_*k*_}) were adjusted to control the FDR for independent test statistics (Benjamini and Hochberg, 1995). Only proteins with an FDR < 0.01 corresponding to *p* values < 0.00026, < 0.00045, < 0.00002, and < 0.000003 were deemed significant for the *Cv*_*i*_, *T_j_*, *Cv*_*i*_ × *T_j_*, and *Cv*_*i*_ × *T_j_* {*S*_*k*_} effects, respectively. Following the methodology described by Kumar and Futschik (2007), fuzzy C-means clustering of wheat proteins showing significant abundance changes according to each effect tested was performed from *Z*-score transformed values and a fuzzification parameter of 2, with the exception of the *Cv* × *T{S}_effect* proteins for which a hierarchical clustering was realized using the Euclidean distance as dissimilarity metric and the Ward’s method as aggregation criteria.

Based on the results of the ANOVA, regularized generalized canonical correlation analysis (rCCA) were used to assess the canonical relationships between the abundance changes of wheat proteins and the accumulation of fungal effector proteins that are supposed to putatively control host biological processes. This rCCA was computed from all wheat proteins harboring an interaction effect of the two main factors (i.e., *Cv* × *T_effect* and *Cv* × *T*{*S*}*_effect* proteins) and the *F. graminearum* putative effectors identified from the same biological samples and described in Fabre et al. (2019a). More specifically, all *F. graminearum* proteins displaying abundance patterns significantly impacted by the host cultivar and/or the fungal genetics have been primarily selected (Fabre et al., 2019a). Among these, only fungal proteins predicted as effector using EffectorP2.0 (Sperschneider et al., 2018) and/or secreted according to the predicted *F. graminearum* secretome described in Brown et al. (2012) were chosen. These structural features used to select the fungal proteins have been extracted from Fabre et al. (2019a) and are provided in Supplementary Table 1. The rCCA was performed following the methodology described in Gonzalez et al. (2008) and using the mixOMICS r-package v5.2 (Rohart et al., 2017). Since the number of subjects was lower than the number of variables in both datasets, the regularization parameters λ_1_ and λ_2_ estimated following the methodology described in Budzinski et al. (2019) were used for the covariance matrices X and Y. The pairwise association matrix was computed for the first eight dimensions, and all canonical correlations have been plotted using the *network* function with a threshold set to 0.95.

### Protein and Gene Ontology Annotations

Functional annotation of all the identified wheat proteins was performed using HMMER 3.2 (June 2018) by comparison of the protein sequences with the HMM PFAM A database 32.0 (February 2019; *p* < 0.01, dom *e*-value < 0.01). The complete results of this analysis are provided in Supplementary Table 2. Only the best result has been kept, and the correspondence between the Gene Ontology (GO) ID and PFAM ID was performed using PFAM2GO mapping (released April 21, 2018) (Mitchell et al., 2015). All GO terms matching the identified wheat proteins can be found in Supplementary Table 3. Gene Ontology enrichment in the different protein clusters was computed using a chi-squared test between the observed and expected protein lists as described in Fabre et al. (2019a). Adjusted *p* values were calculated with the FDR procedure for multiple testing under dependency (Benjamini and Yekutieli, 2001) and deemed significant when < 0.01. Wheat sequences were also compared with the plant susceptibility factors already described and experimentally verified in the literature using a BLAST analysis (best hit *p* < 1 × 10^–80^, identity > 70%).

## Results

In this work, the molecular responses to FHB occurring at 72 hpi were surveyed in three wheat cultivars facing three *F. graminearum* strains of contrasting susceptibilities and aggressiveness, respectively. At this time point, previous analyses performed on the same host and pathogen couples demonstrated the development of a similar fungal mass for the three *F. graminearum* strains regardless of the host, while the symptom severity significantly differed between the three host cultivars, ranking Recital, Cadenza, and Renan in decreasing order of susceptibility (Fabre et al., 2019a).

### Proteomics Profiling of the Three Wheat Cultivars of Contrasting FHB Susceptibilities

**FIGURE 1:**
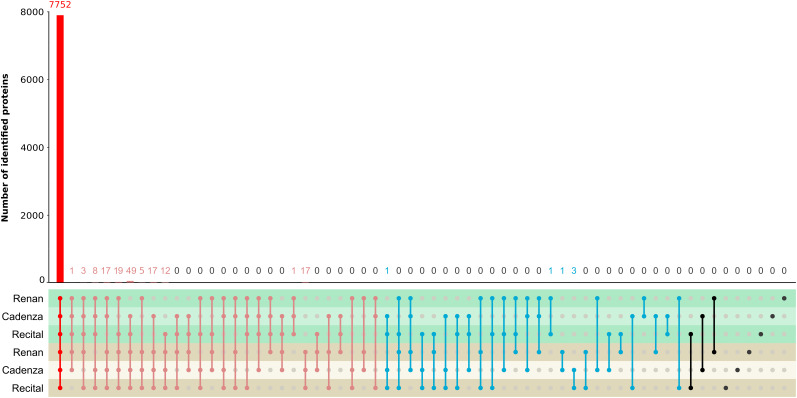
Representation of the number of wheat proteins identified in the different samples. For each infected (in *brown*) and control cultivar (in *green*), the number of proteins identified in each sample combination is indicated in *red color* for the three wheat cultivars’ core proteome, in *light red* for the extended core proteome, in *blue* for the accessory proteome, and in *black* for the specific cultivar proteins.

Considering the nine wheat cultivar × *F. graminearum* strain pairs and the water-inoculated plants, this analysis allowed the identification of 7,907 wheat proteins including about 65% of the proteins already identified in our previous work (Supplementary Table 1; Fabre et al., 2019b). All protein sequences are provided in Supplementary Material 1. Three distinct protein sets have been categorized according to their detection in the different host by fungal strain combinations: a “core proteome” set gathered all common proteins to the three wheat cultivars identified in both infected and control samples; an “extended core proteome” set, determined by analogy to the extended core genome described by Lapierre and Gogarten (2009), was characterized by proteins common to the three cultivars but not necessarily identified in both the infected and control conditions; and an “accessory proteome” set including all proteins undetected in each sample of at least one wheat cultivar (Figure 1). According to such categorization, 7,752 proteins (≈98% of the total identified proteins) belonged to the core proteome, while only 149 proteins accounted for the extended core proteome (Figure 1). Of these extended core proteins, 13 and 136 were found to be strictly common to the three cultivars only under the control and inoculated conditions, respectively (Figure 1 and Supplementary Table 4). Only one protein, an “AMP-binding enzyme,” was specific to the control samples, while 17 others were exclusively identified in the *F. graminearum*-inoculated samples, including one “protease inhibitor,” one “cytochrome P450,” and two “UDP-glucosyl transferase” proteins (Figure 1 and Supplementary Tables 1, 4). Regarding the accessory proteome, only six proteins were found to be systematically absent in at least one cultivar. Among these, one cysteine-rich gliadin protein appeared to be undetected in the Renan samples, one peroxidase was identified in the water-inoculated Renan and Recital samples only, one thaumatin-like protein was detected in the infected Renan and Cadenza plants only, while three proteins (i.e., cysteine-rich gliadin, cytochrome P450, and a protein of unknown function) were found in the infected Cadenza and Recital samples only (Supplementary Tables 1, 4). In addition, none of the identified proteins appeared to be specific to a given wheat cultivar in both the infected and control conditions (Figure 1). Only 12, 17, and five specific proteins were detected in the control samples Recital, Cadenza, and Renan, respectively, but all of them were found in the three cultivars when facing *F. graminearum* infection (Supplementary Table 4).

### Can the Wheat Core Proteome Differentiate the Three Cultivars Through Differential Protein Accumulations?

Quantitative analysis at the individual protein level was performed using ANOVA to evaluate the significance of the different effects on the protein abundance changes between the samples. These tested effects allowed the categorization of the regulated proteins into five groups: (i) the *Cv_effect* proteins that correspond to proteins whose abundance differences are only explained by the wheat genetic background without any effect of *F. graminearum* infection; (ii) the *T_effect* proteins that are characterized by a similar abundance change for the three wheat cultivars in the presence of *F. graminearum* without any variance driven by the wheat genetic backgrounds; (iii) the *Cv* + *T_effect* proteins that refer to proteins displaying similar abundance variations for the three cultivars during the infection process while maintaining the baseline differences discriminating the three wheat genetic backgrounds in the control condition; (iv) the *Cv* × *T_effect*, and (v) the *Cv* × *T{S}_effect* proteins that correspond to proteins distinguishing the different wheat cultivars in their general response to the infection or to a specific *F. graminearum* strain, respectively.

**FIGURE 2:**
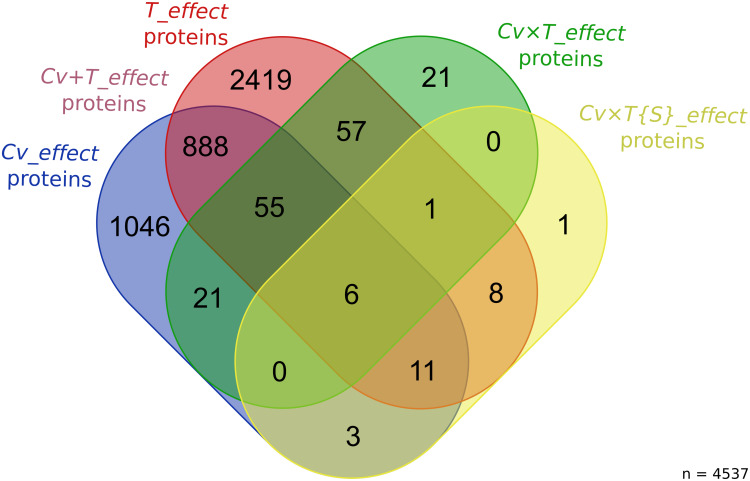
Number of wheat proteins significantly impacted by the different effects tested in the ANOVA. The Venn diagram shows the number of wheat proteins with significant variations in abundance for each factor of the ANOVA [*Cv_effect* proteins: false discovery rate (FDR) < 1%, *p* < 0.00026; *T_effect* proteins: FDR < 1%, *p* < 0.00045; *Cv* × *T_effect* proteins: FDR < 1%, *p* < 0.00002; and *Cv* × *T{S}_effect* proteins: FDR < 1%, *p* < 0.000003].

**FIGURE 3:**
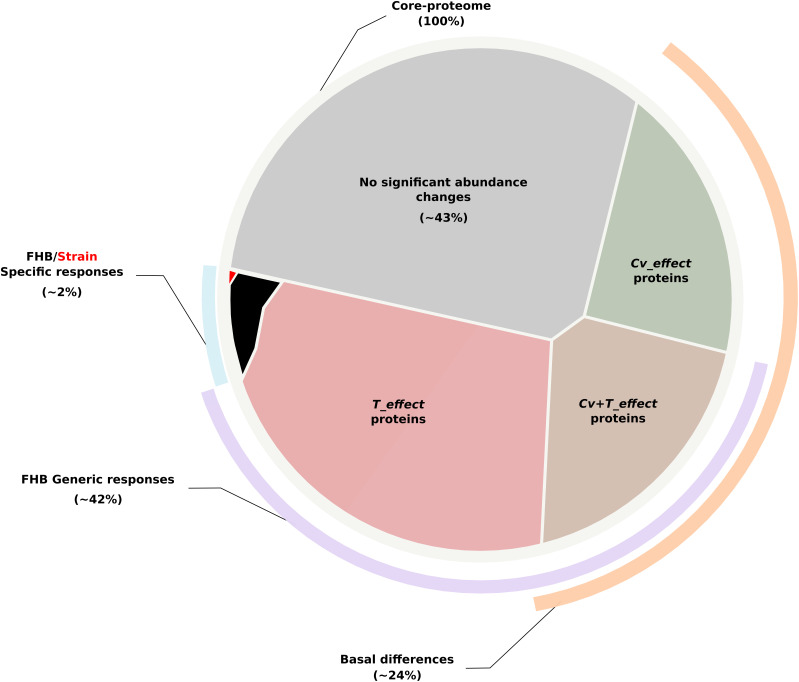
Voronoï representation of the wheat core proteome from the protein abundance patterns. The circular plane, depicting all identified proteins belonging to the core proteome, is partitioned into regions illustrating the proportion of (i) proteins with no significant abundance change according to the factors tested in the ANOVA (in *gray*); (ii) proteins whose abundance differences were only explained by the wheat genetic background (i.e., *Cv_effect* proteins depicted in *green*); (iii) proteins whose abundance differences were specifically explained by the presence of *Fusarium graminearum stricto sensu* (i.e., *T_effect* proteins depicted in *light red*); (iv) proteins displaying significant abundance variations during the infection while maintaining the baseline differences discriminating the three wheat cultivars in the control condition (i.e., *Cv* + *T_effect* proteins depicted in *brown*); and (v) proteins harboring significant abundance variations allowing to distinguish the different wheat cultivars in their response to the disease (i.e., *Cv* × *T_effect* proteins, in *black*) or to a particular *F. graminearum* strain (*Cv* × *T{S}_effect* proteins, in *red*). According to such categorization, *lines outside the circle* show the core proteome proportions corresponding to the basal differences between the three cultivars (in *orange*), the wheat generic responses to the disease (in *purple*), and the wheat genetic-dependent responses to FHB (in *blue*).

As a whole, 4,537 proteins (∼57%) were deemed significant for at least one of the effects tested in the ANOVA (see Supplementary Table 5 for all the protein abundance values). A total of 2,419 *T_effect* proteins, 1,046 *Cv_effect* proteins, 888 *Cv* + *T_effect* proteins, 154 *Cv* × *T_effect* proteins, and 30 *Cv* × *T{S}_effect* proteins were identified (Figure 2 and Supplementary Tables 1, 6). According to such categorization and their overlaps, three protein datasets have been further defined. The first one gathers proteins reflecting the basal abundance differences between the three wheat cultivars (*Cv_effect* and *Cv* + *T_effect* proteins), the second one includes proteins depicting wheat generic responses to FHB (*T_effect* and *Cv* + *T_effect* proteins), and the third gathers proteins characterizing a cultivar- and strain-specific response to the infection (*Cv* × *T_effect* and *Cv* × *T{S}_effect* proteins) (Figure 3).

A primary analysis of the whole set of protein abundance profiles for all the wheat cultivar × *F. graminearum* strain pairs and the control samples using PCA identified two principal components accounting for 39.3% of the total dataset variance (Supplementary Figure 1A). The largest source of variation (Dim 1 = 26.3%) emphasized that the presence of *F. graminearum* is responsible for massive changes in wheat proteomes without any major effect from the genetic background of the inoculated fungal strain. The second PCA component (Dim 2 = 13%), consistent with the dispersion of samples according to their respective FHB susceptibility level, indicated protein abundance differences in the three wheat cultivars without any prominent effect related to the infection. Another PCA score plot using only the protein subset corresponding to the basal abundance differences between the cultivars (i.e., *Cv_effect* and *Cv* + *T_effect* proteins) confirmed the clear separation of the three cultivars, ranked in increasing order of FHB susceptibility on the first axis (Dim 1 = 30.3%) for both the water-inoculated and the *F. graminearum*-inoculated samples (Supplementary Figure 1B).

**FIGURE 4:**
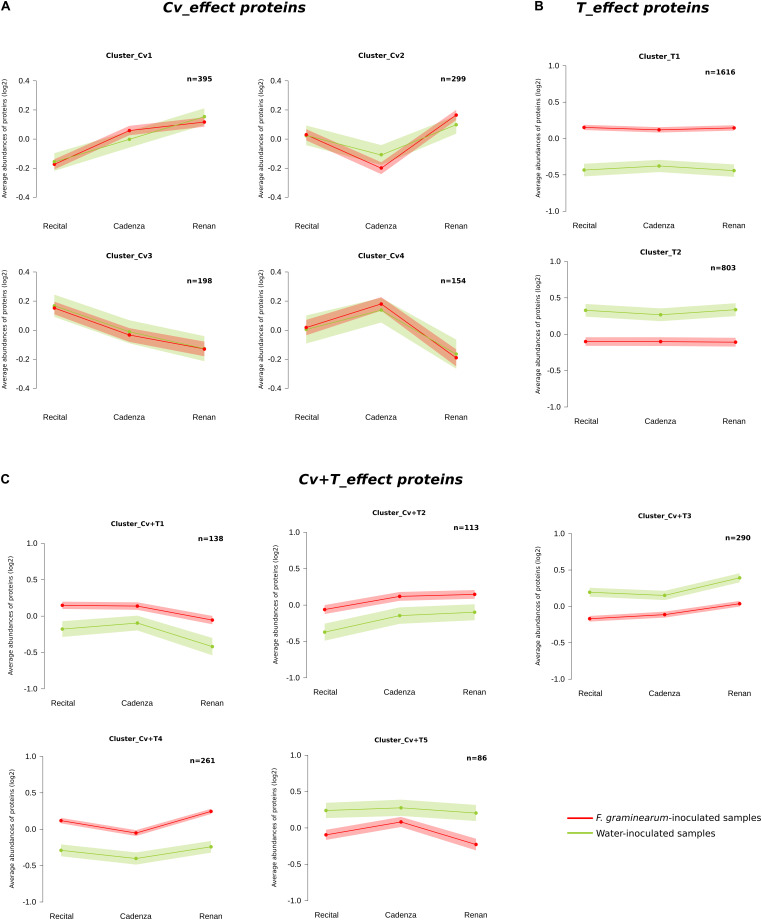
Clustering representation of the wheat proteins displaying basal differences between wheat cultivars and generic responses upon *Fusarium graminearum* inoculation. Clustering was computed using the fuzzy C-means method from *Z*-score transformed values to identify homogeneous patterns in **(A)** *Cv_effect* proteins, **(B)** *Treatment_effect* proteins, and **(C)** *Cultivar* + *Treatment_effect* proteins. For each protein cluster, the average profile was represented by the *line plot* with the wheat cultivars on the *x*-axis and the normalized abundances on the *y*-axis. A color code was given for the two treatment modalities: *red*: average protein abundances observed in *F. graminearum*-inoculated samples; *green*: average protein abundances observed in the water-inoculated samples. The number of proteins in each cluster is indicated in the *upper right-hand corner* of the graphs (*n*). *Ribbons* indicate the 95% confidence interval and retain the same color code.

As a whole, 54% of the differentially accumulated proteins between the three cultivars belonged to the *Cv_effect* proteins. Corresponding to 23% of all identified proteins, the 1,046 *Cv_effect* proteins confirmed significant differences in the protein accumulation between the three cultivars without any effect of *F. graminearum* infection. A fuzzy C-means clustering of the whole set of these proteins evidenced four clusters of consistent abundance profiles (clusters Cv1–Cv4; Figure 4A). They gathered from 154 to 395 proteins accounting for a total of 26 unique significantly enriched molecular functions (Supplementary Table 7). The Cv1 cluster was characterized by protein abundances differentiating the three wheat cultivars according to their respective FHB susceptibilities, with a maximum abundance detected in the Renan samples, an intermediate for Cadenza, and a minimum one for the Recital cultivar. Gene Ontology analysis of the proteins grouped in this cluster highlighted significant enrichments in “chloroplast” proteins (i.e., fold ratio = 44), in “photosystem II oxygen evolving complex” proteins (i.e., fold ratio = 26), and in proteins involved in “proton-transporting ATP synthase activity” (i.e., fold ratio = 32). At the opposite, the Cv3 cluster contained the highest protein abundances in the susceptible cultivar Recital, the intermediate in the Cadenza cultivar, and the lowest ones in the Renan cultivar. In this cluster, the most important functional enrichments were evidenced for proteins involved in “serine-type peptidase activity” (i.e., fold ratio = 39) and in “flavin adenine dinucleotide binding” (i.e., fold ratio = 10). The Cv2 cluster, characterized by higher protein abundances in the cultivars Recital and Renan than in Cadenza, contained significant functional enrichments, of which the prominent ones included proteins participating in “protein refolding” (fold ratio = 114) and in the “large ribosomal subunit” (fold ratio = 51). In the Cv4 cluster, proteins displayed maximum abundances in Cadenza and the lowest in Renan. This group was mostly enriched in proteins acting in carbohydrate metabolism, such as “hydrolase activity, hydrolyzing *O*-glycosyl compounds” (i.e., fold ratio = 16) and “carbohydrate binding” (i.e., fold ratio = 12).

### Are the Responses of Wheat Cultivars to Different Fungal Strains Characterized by a Common Proteomic Signature?

In accordance with the PCA, the ANOVA tests confirmed that the “treatment” factor (i.e., the fungal inoculation) was the largest contributor to the observed abundance differences within wheat proteomes. As a whole, 3,491 proteins (≈77% of the differentially regulated proteins) exhibited abundance variations explained at least in part by the fungal inoculation (i.e., *T_effect*, *Cv* + *T_effect*, *Cv* × *T_effect*, and *Cv* × *T*{*S*}*_effect* proteins) (Figure 2); these included about 50% of the FHB-responsive proteins we already evidenced in our previous analysis of a 96-h-long infection dynamics (Supplementary Table 1; Fabre et al., 2019b). Among the whole set of FHB-responsive proteins in this study, those that did not depend on the genetic background of either member of the pathosystem can be defined as generic wheat responses to infection. They included: (i) *T_effect* proteins, which correspond to proteins with similar abundance variations explained by the presence of the pathogen, whatever the genetic background of the inoculated fungal strains and the wheat cultivars, and (ii) *Cv* + *T_effect* proteins, which could be typified by proteins displaying significant abundance variations in the presence of *F. graminearum* while maintaining the baseline differences discriminating the three wheat cultivars.

The *T_effect* proteins were split into two consistent clusters: the first one included 1,616 proteins whose abundance was significantly higher in the infected samples as compared to the controls (i.e., Cluster_T1; Figure 4B), while the second contained 803 proteins whose abundance decreased significantly during the infection (i.e., Cluster_T2; Figure 4B). The analysis of these two clusters led to the identification of 62 and 31 significantly enriched molecular functions in clusters T1 and T2, respectively (Supplementary Table 7). In Cluster_T1, the strongest enrichments included “malic enzyme activity” and “malate dehydrogenase (decarboxylating) (NAD +) activity” (i.e., fold ratio > 44) and, to a lesser extent, the “chitinase activity” (i.e., fold ratio = 7), while the “fructose-bisphosphate aldolase activity” characterized Cluster_T2 (i.e., fold ratio = 51). Regarding the *Cv* + *T_effect* proteins, a fuzzy C-means clustering of the whole set of these proteins revealed five consistent clusters (Cv + T1 to Cv + T5), gathering from 86 to 290 wheat proteins (Figure 4C). Clusters Cv + T1, Cv + T2, and Cv + T4, grouping a total of 562 proteins, were characterized by higher protein abundances in the *F. graminearum*-inoculated samples as compared to the controls, with maximum abundances for Recital and Cadenza (Cv + T1), for Cadenza and Renan (Cv + T2), or for Renan only (Cv + T4). The strongest enrichments concerned “FMN binding” (fold ratio = 67) in cluster Cv + T1, “phosphopyruvate hydratase activity” (fold ratio = 605) in cluster Cv + T2, and “3-hydroxyacyl-CoA dehydrogenase activity” (fold ratio = 215) in cluster Cv + T4 (Supplementary Table 7). The 376 proteins grouped into clusters Cv + T3 and Cv + T5 displayed lower abundances in the infected samples as compared to the controls, with minimal abundances for Recital and for Renan, respectively. Cluster Cv + T3 was characterized by proteins involved in energy-related processes, such as “chlorophyll binding,” “photosynthetic electron transport chain,” and “photosynthesis, light reaction” (fold ratio > 22), while cluster Cv + T5 was enriched with proteins belonging to “oxidoreductase activity” (fold ratio = 8.6) (Supplementary Table 7).

### Do Wheat Cultivars Set Up Specific Responses to Fusarium Head Blight?

Along with the generic responses to FHB, subtle adjustments in the wheat proteomes also revealed genetic-dependent responses to the disease or to a specific *F. graminearum* strain. A PCA performed using the protein subset corresponding to these specific responses (*Cv* × *T_effect* and *Cv* × *T*{*S*}*_effect* proteins) separated the samples according to the treatment factor on Dim 1 (39.7%; Supplementary Figure 1C). The sample dispersion on Dim 2 revealed that, in the control conditions, the protein profiles were clearly discriminating the three cultivars, while in response to any fungal strain, the regulated protein profiles proved to be closer together. A clear orthogonality is still observed between the cultivars in the control and in response to *F. graminearum* emphasizing the cultivar-specific responses (Supplementary Figure 1C).

**FIGURE 5:**
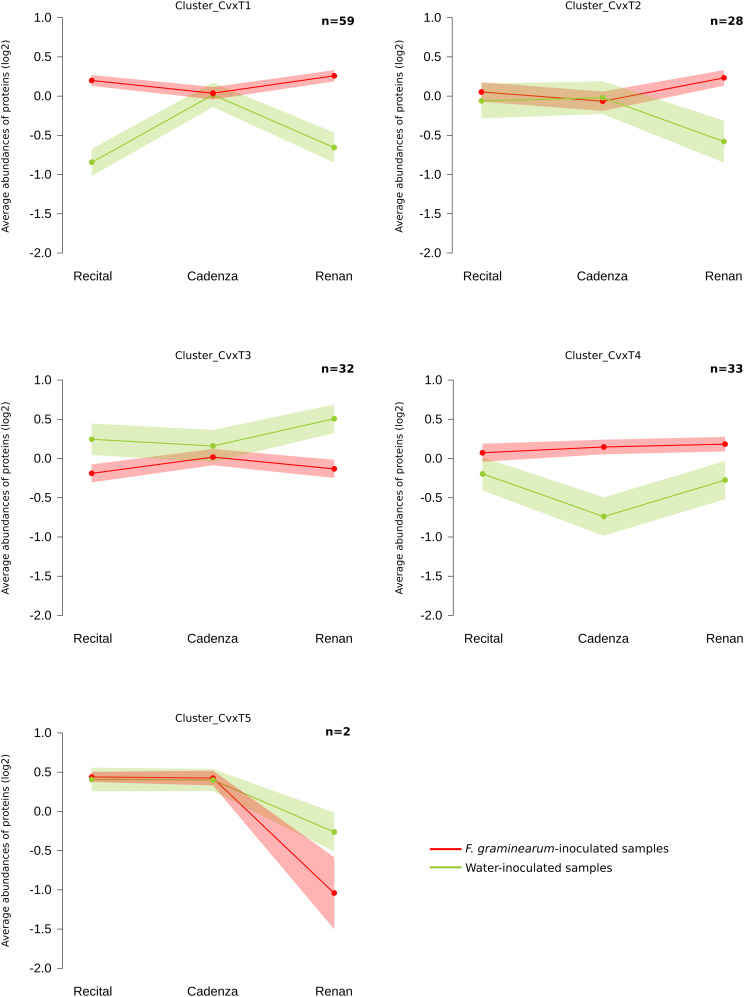
Clustering of wheat *Cultivar* × *Treatment_effect* protein abundance patterns. Clustering was computed using the fuzzy C-means method from *Z*-score transformed values to identify homogeneous patterns. For the five clusters, the average profile was represented by the *line plot* with the wheat cultivars on the *x*-axis and the normalized abundances on the *y*-axis. A color code was given for the two treatment modalities: *red*: average protein abundances observed in *Fusarium graminearum*-inoculated samples; *green*: average protein abundances observed in the water-inoculated samples. The number of proteins in each cluster is indicated in the *upper right-hand corner* of the graphs (*n*). *Ribbons* indicate the 95% confidence interval and retain the same color code.

**FIGURE 6:**
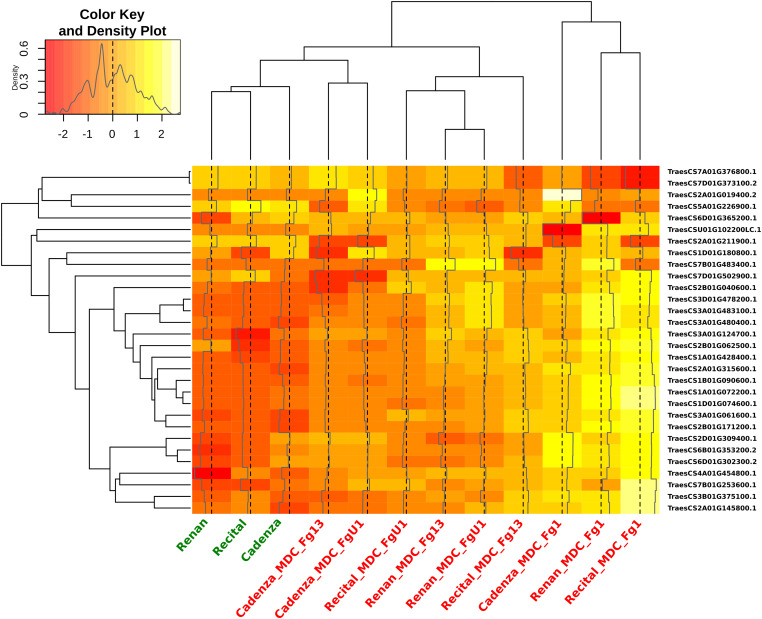
Heat map indicating the abundance changes of the 30 wheat proteins showing significant abundance changes according to the interaction between the wheat cultivar and the inoculated fungal strain. For each cultivar × *Fusarium graminearum* strain combination (in *red*) and control sample (in *green*), hierarchical clustering was calculated from each protein *Z*-score transformed abundance values. The colors of the heat map indicate the abundance of each protein in each sample.

Out of all the proteins harboring significant abundance variations during the infection process, 154 *Cv* × *T_effect* proteins (i.e., 2% of the total quantified proteins) distinguished the different wheat cultivars in their response to the disease. These proteins were characterized by a differential abundance variation between wheat cultivars in the presence of *F. graminearum* without distinction of the inoculated strain. Within this *Cv* × *T_effect* protein set, different protein abundance patterns have been observed, making possible their grouping into five homogeneous clusters (clusters Cv × T1 to Cv × T5) containing from two to 59 proteins (Figure 5). The Cv × T1 group included proteins whose abundances increased significantly in the presence of *F. graminearum* for Recital and Renan cultivars, while no response was noted in Cadenza. The Cv × T2 and Cv × T5 clusters contained proteins whose abundances were especially changed in the Renan cultivar only. The Cv × T3 cluster corresponded to proteins with decreasing abundances in response to *F. graminearum* in the Recital and Renan cultivars only. The Cv × T4 cluster included proteins showing significantly lower abundances in the control samples of the Cadenza cultivar as compared to the other cultivars, while no significant abundance differences could be observed between the three cultivars in the infected samples. In addition, ANOVA allowed the identification of 30 *Cv* × *T{S}_effect* proteins showing significant abundance changes according to the interaction between the wheat cultivars and the inoculated fungal strains. In order to characterize how the genetic background of the *F. graminearum* strain determined the different wheat cultivar responses, a hierarchical clustering of each sample was computed using the abundance patterns of these 30 proteins (Figure 6). This clustering showed two main groups: the first one contained all samples inoculated with MDC_Fg1, the most aggressive *F. graminearum* strain, while the second gathered all the other samples. In this second group, the control samples were closely related to the samples inoculated using MDC_Fg13 and MDC_FgU1, the two less aggressive strains. The functional analysis of these 30 proteins allowed the identification of 18 unique protein functions (Supplementary Table 1), including three cytochrome P450, four aminotransferase class III, two UDP-glucosyl transferases, three glycosyl hydrolases, and two thi4 family proteins.

**FIGURE 7:**
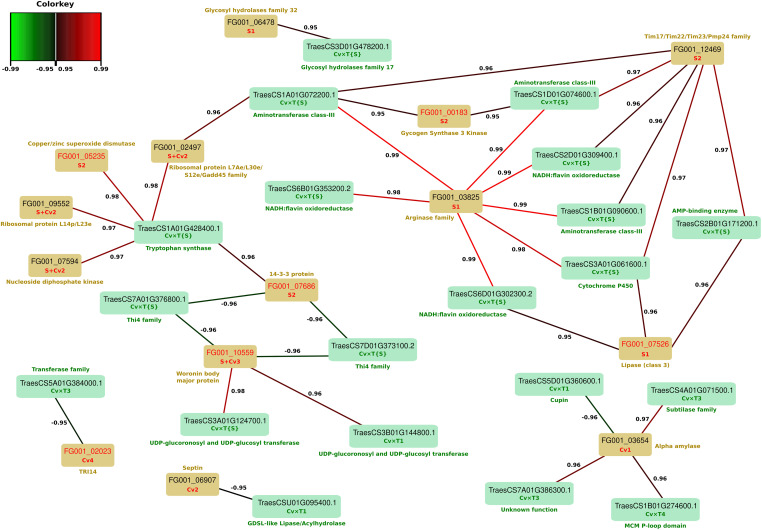
Network plot outlining regularized generalized canonical correlation analysis (rCCA)-derived correlations from wheat proteins and *Fusarium graminearum* putative effectors. Wheat and *F. graminearum* protein accessions are indicated in the *green* and *yellow rectangles*, respectively. *F. graminearum red* accessions highlight the proteins described in the literature to be involved in fungal pathogenicity. For each protein included in the network, the cluster reflecting its pattern of abundance variation is indicated in *green* for wheat proteins and in *red* for *F. graminearum* proteins according to Fabre et al. (2019a). The canonical correlation coefficient derived from the rCCA is represented as a *line between each of the proteins* (negative correlations less than –0.95 in *green* and positive correlations >0.95 in *red*). Predicted protein functions are indicated in *green* and *yellow* for wheat and *F. graminearum* proteins, respectively.

In a previous work realized on the same fungal strains grown on the same wheat cultivars, we already reported a range of fungal protein abundance changes driven by strain genetics and host genotypes (Fabre et al., 2019a). In order to further explore the cultivar-specific responses to FHB infection and to identify their potential links with fungal adjustments, a rCCA was carried out taking into account the fungal protein information in order to identify canonical correlations between the observed wheat protein abundance changes and the *F. graminearum* putative effectors discriminating fungal strains or differentially accumulated in the three wheat cultivars. A total of 79 differentially accumulated putative fungal effectors and 184 wheat proteins (i.e., 154 *Cv* × *T_effect* and 30 *Cv* × *T{S}_effect*) were collected and analyzed. The correlation circle plot and unit representation obtained from this analysis are provided in Supplementary Figures 2, 3. The unit plot shows a clear separation of the samples inoculated with the most aggressive strain, MDC_Fg1 (Dimension XY1), as well as according to wheat genetics (Dimension XY2), suggesting that the different wheat cultivar × *F. graminearum* strain pairs are characterized by distinct protein sets harboring correlated abundance changes. More specifically, all canonical correlation scores between the wheat and *F. graminearum* protein pairs are indicated in Supplementary Table 8. Among the 32,936 calculated scores, 1,079 appeared to be higher than 0.8. Thus, in order to focus on the sharpest relationships, a network plot has been computed from the strongest canonical correlations only (i.e., threshold score = 0.95; Figure 7). Based on this criterion, a total of 20 wheat proteins and 14 putative effectors of *F. graminearum* showed significant canonical correlations with regard to their respective abundance patterns. Except for three fungal proteins (i.e., FG001_02023, FG001_03654, and FG001_06907) whose abundance variances were only explained by the wheat genetic backgrounds, all the other *F. graminearum* putative effectors identified in the network exhibited higher abundances in the most aggressive strain, MDC_Fg1, than in the other two strains (Fabre et al., 2019a). Three putative effectors displayed high covariance scores with a large number of wheat proteins. FG001_03825, belonging to the arginase family proteins, displayed abundance changes strongly associated with seven wheat *Cv* × *T{S}_effect* proteins, including three NADH:flavin oxydoreductases, three aminotransferase class III, and one cytochrome P450. Five of these wheat proteins further appeared to be correlated with the fungal Woronin body major protein FG001_12469, which also showed an abundance pattern close to the wheat AMP-binding enzyme TraesCS2B01G171200.1. In addition, FG001_10559 was positively correlated with two *Cv* × *T{S}_effect* UDP-glucosyl transferases (i.e., TraesCS3A01G124700.1 and TraesCS3B01G144800.1) and negatively correlated with two *Cv* × *T{S}_effect* Thi4 proteins (i.e., TraesCS7A01G376800.1 and TraesCS7D01G373100.2).

## Discussion

### The Bread Wheat–*F. graminearum* Interaction Is Based on a Core Dual Proteome

In this study, mapping the proteome of three wheat cultivars of contrasting susceptibilities did not evidence significant qualitative differences based on the presence/absence of cultivar-specific proteins. Out of a total of nearly 7,910 wheat proteins, only 155 were not identified in all the samples, most of them depicting specific changes in response to the FHB rather than between wheat cultivars. Only a few identified proteins allowed discriminating one particular cultivar in a specific condition. As an example, two proteins were identified in all the infected samples, except in the ones from the most susceptible cultivar, Recital. The first, TraesCS3A01G461100.1, is homologous to the rice SDH4 protein (Q942X4), which is described as a significant source for mitochondrial reactive oxygen species (ROS) production by eliciting part of the salicylic acid (SA)-dependent transcriptional response in plant cells (Jardim-Messeder et al., 2015; Belt et al., 2017; Huang et al., 2019). The second one, TraesCS7B01G483400.1, corresponding to the thaumatin-like PWIR2 protein (P27357), has already been shown to be involved in fungal resistance in wheat (Rebmann et al., 1991; Andersen et al., 2018). Although the specific absence in the infected Recital samples of these few proteins appeared to be consistent with its observed susceptibility, our results indicate that approximately 98% of the identified proteins were detected in all the analyzed samples. This emphasizes that similar wheat proteomes are set up despite large differences in FHB susceptibility. Echoing previous findings reporting that different strains produce also very similar protein contents (Fabre et al., 2019a), this suggests that the bread wheat–*F. graminearum* interaction involves a conserved protein set with marginal genetic specificities. Although similar, this dual proteome is nonetheless quantitatively variable, as demonstrated by the number of wheat protein abundance changes. Though the study should be extended to more genotypes, this assumes further that wheat resistance/susceptibility to FHB and *F. graminearum* aggressiveness could be mainly based on quantitative molecular mechanisms.

### A Common Base of Disease-Responsive Proteins Is Regulated Regardless of Wheat Susceptibility to FHB

Among all the proteins differentially accumulated in response to the *F. graminearum* inoculation, nearly 95% did not exhibit abundance variations, allowing to distinguish the three cultivars in their responses to infection (i.e., *T_effect* and *Cv* + *T_effect* proteins). A wide range of these proteins was involved in defense, thus depicting the implementation of basal mechanisms in response to FHB shared by the three cultivars. These include known regular processes involved in the response to fungal pathogen, such as several chitinase proteins involved in fungal cell wall disruption (Jain and Khurana, 2018), as well as more atypical ones such as the ubiquitin-like modifier activating enzymes whose systemic role can modulate immune responses (He et al., 2017; Skelly et al., 2019). The most prominent functional enrichment found in these common responses concerned the NAD- and NADP-dependent malic enzymes, whose involvement in disease resistance has been reported as well through ROS synthesis (Legendre et al., 1993; Chi et al., 2009; Park et al., 2013; Heng et al., 2020). Similarly, wheat proteins involved in phosphatidylcholine metabolic process showed large increases in abundance in all three wheat cultivars in response to infection. Phosphatidylcholine is involved in signal transduction between stress-responsive membrane enzymes in order to elicit vacuolar proton fluxes that trigger the phytoalexin response after the detection of fungal elicitors (Viehweger et al., 2002; Zhao, 2015). Along with the few fungal strain-dependent responses, these results emphasize that most of the qualitative and quantitative molecular adjustments induced by the infection are not only similar in the three wheat cultivars but also appear to be unrelated to the aggressiveness of the *F. graminearum* strain.

An additional search for the known susceptibility genes among our dataset evidenced 17 homolog candidates (Supplementary Table 9), including 14 that displayed substantial accumulations in response to FHB regardless of the host and the pathogen genetic backgrounds. Two of these proteins encoded by wheat genes homologous to *AtFER* from *Arabidopsis thaliana* were identified in this work (i.e., TraesCS1B01G241400.1 and TraesCS4B01G171100.1). While the role of these genes is not yet fully understood in wheat (Wood et al., 2020), it was shown that an *A. thaliana* defective mutant in FERONIA displayed enhanced resistance to *Fusarium oxysporum* and to powdery mildew infection (Kessler et al., 2010; Masachis et al., 2016; Fernandes et al., 2017). A similar increase in protein abundance was also identified for five other proteins harboring a high degree of homology with the proteins encoded by susceptibility genes known to be involved in the inhibition of the salicylic acid biosynthesis pathway (Supplementary Table 9). Two of these proteins, TraesCS3A01G426800.3 and TraesCS7A01G304000.1, homologous to OsSTAD2 and TaMDAR6, respectively, belong to the chloroplast compartment (Jiang et al., 2009; Abou-Attia et al., 2016; Yang et al., 2016; Yu et al., 2017), providing further evidence of the putative role of this organelle in FHB susceptibility as a pivotal target of fungal manipulation processes (Fabre et al., 2019b). In addition, two other proteins (TraesCS1A01G350500.1 and TraesCS3A01G282800.1), respectively known as TaSnRK1α1-A and TaSnRK1α2-A, were identified to be similarly downregulated in the three cultivars facing FHB. Previously shown to be involved in *F. graminearum* toxin tolerance (Perochon et al., 2019), TaSnRK1α proteins are also known to be targeted by the fungal OSP24 effector in order to trigger their degradation through the SCF (Skp–Cullin–F-box) ubiquitin ligase complex and the 26S proteasome (Jiang et al., 2020). The observed abundance decrease of these two proteins could suggest a fungal manipulation of this defense process that enhances wheat susceptibility independently of the plant and fungal genetics. Although the study of only three wheat cultivars with three *F. graminearum* strains is not sufficient to generalize these observations, the absence of specific responses in these candidate susceptibility genes echoes the already stated hypothesis of Hales et al. (2020) suggesting that the wheat susceptibility to FHB could be common to a wide range of current cultivars. Our results further complement this hypothesis by suggesting that the molecular determinants of FHB susceptibility may also be unspecific to the fungal strain.

### Marginal Proteome Regulations Discriminate Wheat Cultivars of Contrasting FHB Susceptibilities

In this work, only 5% of all the FHB-regulated proteins were able to differentiate the three cultivars in their responses to the disease. With the exception of the 30 proteins of the Cv × T2 and Cv × T5 clusters unassigned to a particular biological process or molecular function, these cultivar-specific responses mainly reflect a rebalancing of the basal differences observed in the control samples during the infection process. Although these singular abundance variation patterns demonstrate a broader plasticity in the proteome adjustments of a given cultivar, their final accumulation does not especially discriminate the three wheat cultivars. Moreover, only 30 FHB-regulated wheat proteins displayed abundance changes driven at least in part by the strain genetics (i.e., *Cv* × *T{S}_effect* proteins). The additional power provided by the integrative analysis of both the host and the pathogen datasets identified covariance patterns between 13 wheat *Cv* × *T{S}_effect* proteins and 10 putative *F. graminearum* effectors, among which five were previously described to be involved in fungal virulence: FG001_05235, FG001_07686, FG001_07526, FG001_00183, and FG001_10559 (Markham and Collinge, 1987; Voigt et al., 2005; Niu et al., 2013; Son et al., 2013; Blümke et al., 2014; Qin et al., 2015; Yao et al., 2016; Furukawa et al., 2017; Brauer et al., 2019a; Fernando et al., 2019). Four of these fungal proteins exhibited abundance changes explained only by the *F. graminearum* genetic backgrounds, with a maximal abundance detected in the samples inoculated with the most aggressive strain, MDC_Fg1 (Fabre et al., 2019a). Although these 13 wheat proteins could be direct or indirect targets of these fungal proteins, the finding that the *Cv* × *T{S}_effect* protein profiles were mainly correlated with fungal *S_effect* proteins indicated that the observed effect is primarily due to the fungal genetics rather than to the differences between cultivars. This assumption is supported by the weak detection of canonical correlations between the fungal proteins and the wheat *Cv* × *T_effect* proteins. Despite the detection of different molecular functions already shown to be involved in plant defense, such as UDP-glucosyltransferases (Pasquet et al., 2016; He et al., 2020) and the Thi4 protein family (Yusof, 2019), the marginal detection of such patterns of abundance changes suggests that the impact of the infection and/or fungal strain genetics has only a specific and a limited effect on the host responses.

### Basal Proteome Differences Could Drive FHB Susceptibility in Wheat

Although a limited number of protein regulations illustrated cultivar-specific responses to infection, a significant proportion of plant proteins showed basal differences that could explain the observed contrasts in disease severity. Overall, 46.7% of the proteins displayed significant abundance changes driven at least in part by the cultivar genetics, among which, chloroplast functions are particularly discriminating. Chloroplast plays an essential role in the biosynthetic pathways of phytohormones involved in the range of defense mechanisms set up by the plant cell (Sowden et al., 2017; Lu and Yao, 2018), and they communicate with other organelles to regulate the expressions of several nuclear defense genes (Shapiguzov et al., 2012; Sierla et al., 2013). In addition, several studies have already shown that chloroplast processes were particularly targeted by fungal effectors (de Torres Zabala et al., 2015; Petre et al., 2015, 2016; Lorrain et al., 2018; Fabre et al., 2019b; Han and Kahmann, 2019; Kretschmer et al., 2019), suggesting that the control of chloroplast by pathogens could be a key milestone for the infection success. In this work, chloroplast proteins discriminating wheat genotypes were systematically more abundant in the less susceptible cultivar Renan than in the other two. These include (i) proteins of the photosystem II oxygen evolving complex, which did not show abundance changes in the presence of *F. graminearum* (i.e., cluster Cv1), and (ii) proteins involved in “chlorophyll binding,” “photosynthetic electron chain,” and “photosynthesis” functions identified to be downregulated during the infection process while maintaining the differences observed in the control samples. This corroborates a previous work that demonstrated a role of photosynthesis adjustments in FHB resistance (Francesconi and Balestra, 2020) and further suggests that part of the observed differences in disease severity could not be explained only by differential magnitudes in the cultivar responses to infection but rather by the abundance of proteins that basically distinguish the three wheat cultivars under optimal conditions. The same statement can be put forward for the different proteins known to be involved in plant defense processes, such as Wheatwin proteins (TraesCS3D01G524700.1 and TraesCS3B01G584700.1, clusters Cv1 and Cv + T3, respectively) displaying antifungal activity (Caporale et al., 2004; Bertini et al., 2009). These two proteins showed the highest abundances in the less susceptible cv. Renan, as well as four other proteins (TraesCS3B01G362600.1 in Cv1 and TraesCS5A01G226400.1, TraesCS5B01G416700.1, and TraesCS6A01G059600.1 in Cv + T4) coded by genes previously identified to be localized in high-confidence meta-QTL regions involved in wheat resistance to FHB (Zheng et al., 2020). This assumption can also be argued by the three homologs of candidate susceptibility genes showing differences in abundance between wheat cultivars (Supplementary Table 9). Two proteins (TraesCS4A01G202100.2 and TraesCS4B01G106300.1, cluster Cv + T1), closely related to the barley ADH1 known to contribute to the nourishment of *Blumeria graminis* (Pathuri et al., 2011; van Schie and Takken, 2014), harbored systematically the lowest abundance in all the Renan samples. These two proteins showed similar abundance increases for all three cultivars in response to infection while maintaining the abundance differences already observed in the control samples. Likewise, the TraesCS4D01G267600.2 protein (cluster Cv3) homologous to the *A. thaliana* CD48A described as a negative regulator of nucleotide-binding leucine-rich repeat (NLR)-mediated immunity (Copeland et al., 2016), did not respond to *F. graminearum* inoculation, but displayed cultivar-specific accumulations that fit with their respective susceptibility levels. Taken together, such basal contrasts in protein abundances could indirectly contribute to the observed susceptibility levels of the three cultivars and suggest that some FHB molecular determinants derive from the basal differences between wheat genetic backgrounds that are maintained during the infection rather than through cultivar-specific responses to the infection.

## Conclusion

**FIGURE 8:**
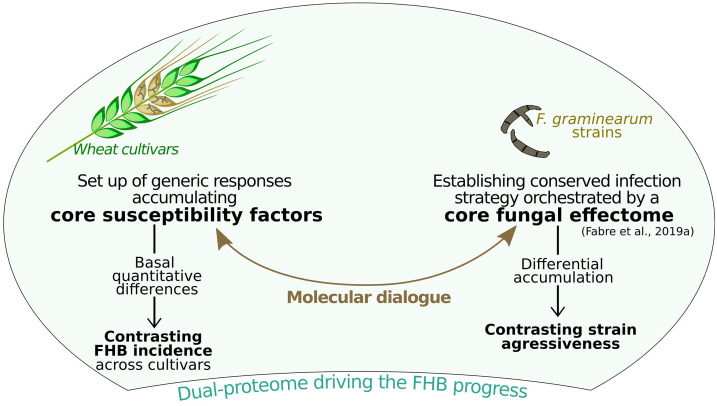
Working model summarizing the core dual proteome hypothesis between wheat and *Fusarium graminearum*. Wheat spike was obtained from https://www.freepik.com.

Although qualitatively similar, the plant-responsive patterns to *F. graminearum* strains proved to be quantitatively variable through differentially accumulated proteins. Overall, most of the identified protein regulations did not clearly distinguish between the three cultivars in their responses to infection, while a number of basal differences in common processes can be linked to their respective FHB susceptibility (Figure 8). Linking all these results with those previously described on the fungal side (Fabre et al., 2019a), this study depicts that the interaction is driven by the setup of a core dual proteome shaping similar infection strategies in different strains to manipulate very close cellular processes in different wheat genetic backgrounds. This supports the hypothesis that aggressiveness and susceptibility are respectively intrinsic characteristics of *F. graminearum* strains and wheat cultivars and that they are, moreover, independent of each other. Although additional analyses using more wheat and *F. graminearum* genetic backgrounds will be required to validate such assumptions, acting on wheat cultivar common processes involved in FHB susceptibility could be of considerable value to improve FHB resistance, whose efficiency would not be balanced by the usual evolvement of fungal strains.

## Data Availability Statement

The mass spectrometry proteomics data have been deposited to the ProteomeXchange Consortium (Deutsch et al., 2020) via the PRIDE (Vizcaíno et al., 2016) partner repository with the dataset identifiers PXD023212 and PXD015139.

## Author Contributions

TL and LB designed the research. FF, SR, and LB prepared the samples. FF, SU, and LB performed proteomic experimentation. FF and LB conceived and performed the modeling, analyzed the data, prepared the figures, and wrote the manuscript. All authors contributed to the article and approved the submitted version.

## Conflict of Interest

The authors declare that the research was conducted in the absence of any commercial or financial relationships that could be construed as a potential conflict of interest.

## Footnotes

1 https://www.thegpm.org/TANDEM/release.html

2 https://plants.ensembl.org/Triticum_aestivum/Info/Index

## Supplementary Material

Supplementary Figure 1 Representation of the sample dispersion from the principal component analysis. For each biological replicate of the cultivar × strain combinations and the control samples, principal component analysis was performed using: **(A)** the whole set of identified wheat protein quantification values, **(B)** a subset of protein corresponding to the basal abundance differences between the three wheat cultivars (*Cv_effect* and *Cv* + *Cv_effect* proteins) and, **(C)** a subset of protein quantification values reflecting a cultivar-specific response to the disease (*Cv* × *T_effect* proteins) or *F. graminearum* strain-specific adjustments (*Cv* × *T{S}_effect* proteins). Water-inoculated samples are indicated in light blue, light green and light red for Recital, Cadenza, and Renan cultivars respectively. *F. graminearum*-inoculated samples were plotted in blue, green and red for Recital, Cadenza, and Renan cultivars respectively. For each Cultivar × Treatment groups, 95% confidence ellipses were plotted using the same color code.

Supplementary Figure 2 rCCA correlation circle plot from the two first components for all wheat cultivar – *F. graminearum* strain combinations. Blue triangles correspond to the wheat proteins and Red circle to the *F. graminearum* putative effectors identified in Fabre et al. (2019a).

Supplementary Figure 3 rCCA unit representation plot from the two first components for all wheat cultivar – *F. graminearum* strain combinations.

Supplementary Table 1 Table summarizing bioinformatics analyses of the identified wheat proteins and *F. graminearum* putative effectors.

Supplementary Table 2 Functional annotations of the identified wheat proteins using HMMER and PFAM-A database.

Supplementary Table 3 Gene ontology annotations of all identified wheat proteins.

Supplementary Table 4 Summary table indicating the presence/absence analysis of the wheat proteins identified in the different samples.

Supplementary Table 5 Table showing the average of wheat protein abundances quantified in the three biological replicates for each cultivar × *F. graminearum* strain combination and control samples.

Supplementary Table 6 List of the protein groups described in the Venn diagram.

Supplementary Table 7 Gene ontology enrichment identified in wheat protein clusters.

Supplementary Table 8 Table indicating all canonical correlation scores between wheat and *F. graminearum* protein pairs.

Supplementary Table 9 Table indicating the potential susceptibility factors identified from literature and corresponding to the wheat proteins identified in this work.

Supplementary Material 1 Fasta file containing all wheat protein sequences identified in this work.

## Abbreviations

ANOVA: analysis of variance
DON: deoxynivalenol
FHB: Fusarium head blight
FDR: false discovery rate
hpi: hours post-inoculation
QTL: quantitative trait loci
*Cv*: cultivar effect
*T*: treatment effect
*Cv* + *T*: cultivar + treatment effect
*Cv* × *T*: cultivar × treatment effect
*Cv* × *T{S}*: cultivar × strain effect
rCCA: regularized generalized canonical correlation analysis.
