# Supplementary figures and images for: Proteomics-Based Data Integration of Wheat Cultivars Facing Fusarium graminearum Strains Revealed a Core-Responsive Pattern Controlling Fusarium Head Blight
Source: Front Plant Sci. 2021 May 31;12:644810. doi: 10.3389/fpls.2021.644810 (PMC8201412; doi:10.3389/fpls.2021.644810)

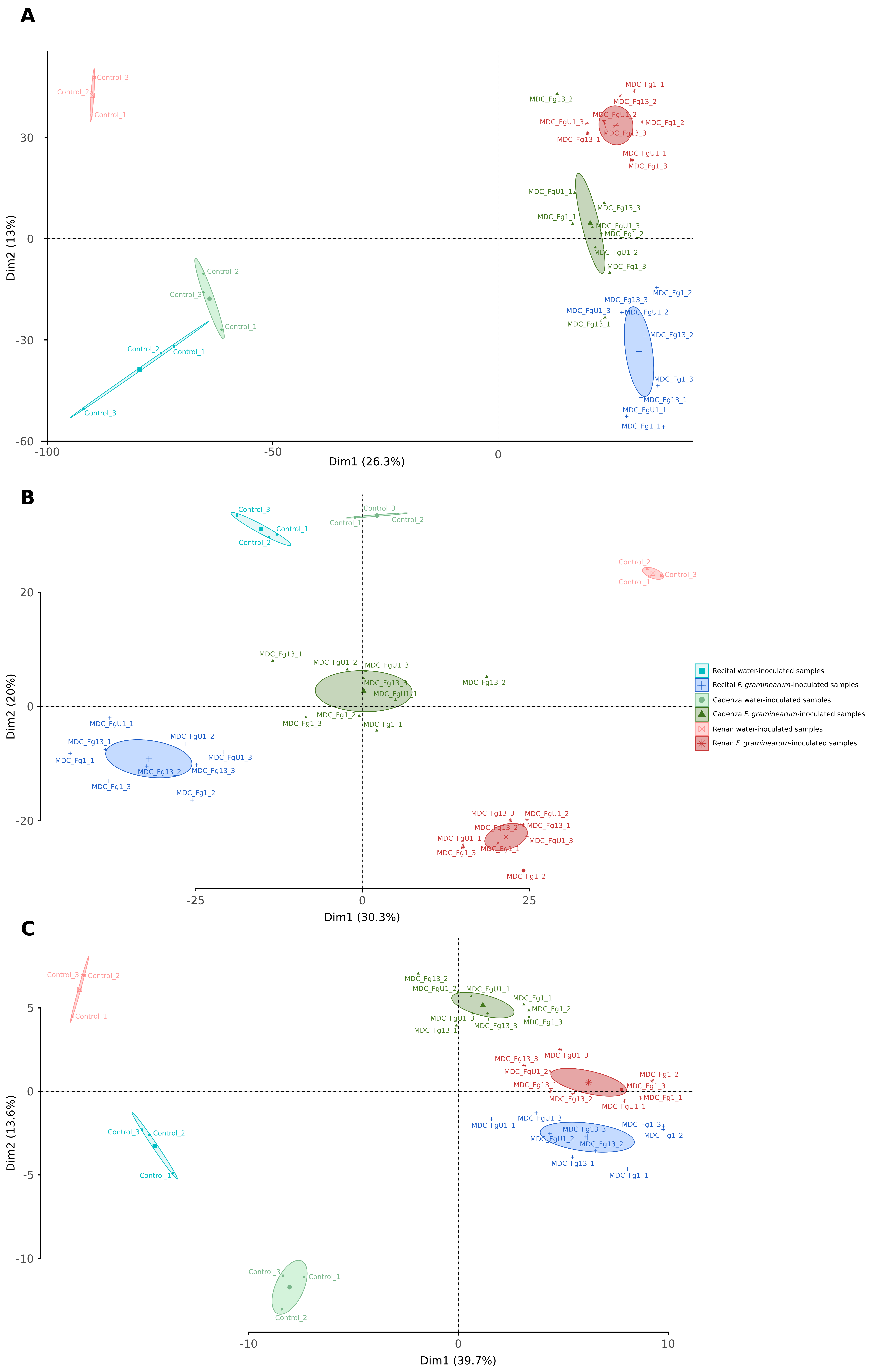

Supplement: Supplementary Figure 1 — Representation of the sample dispersion from the principal component analysis. For each biological replicate of the cultivar × strain combinations and the control samples, principal component analysis was performed using: (A) the whole set of identified wheat protein quantification values, (B) a subset of protein corresponding to the basal abundance differences between the three wheat cultivars (Cv_effect and Cv + Cv_effect proteins) and, (C) a subset of protein quantification values reflecting a cultivar-specific response to the disease (Cv × T_effect proteins) or F. graminearum strain-specific adjustments (Cv × T{S}_effect proteins). Water-inoculated samples are indicated in light blue, light green and light red for Recital, Cadenza, and Renan cultivars respectively. F. graminearum-inoculated samples were plotted in blue, green and red for Recital, Cadenza, and Renan cultivars respectively. For each Cultivar × Treatment groups, 95% confidence ellipses were plotted using the same color code. [file Image_1.TIFF]

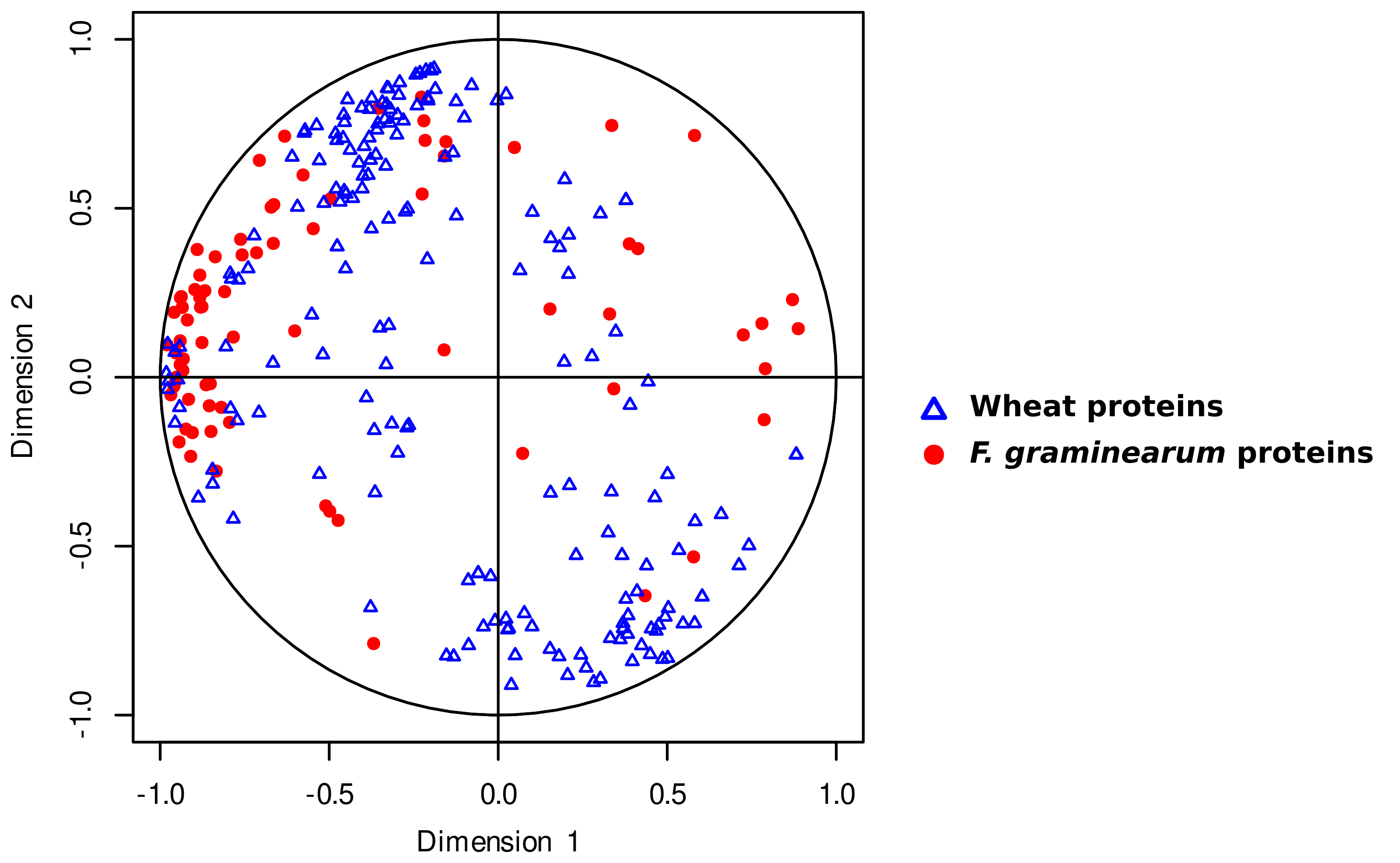

Supplement: Supplementary Figure 2 — rCCA correlation circle plot from the two first components for all wheat cultivar – F. graminearum strain combinations. Blue triangles correspond to the wheat proteins and Red circle to the F. graminearum putative effectors identified in Fabre et al. (2019a). [file Image_2.TIFF]

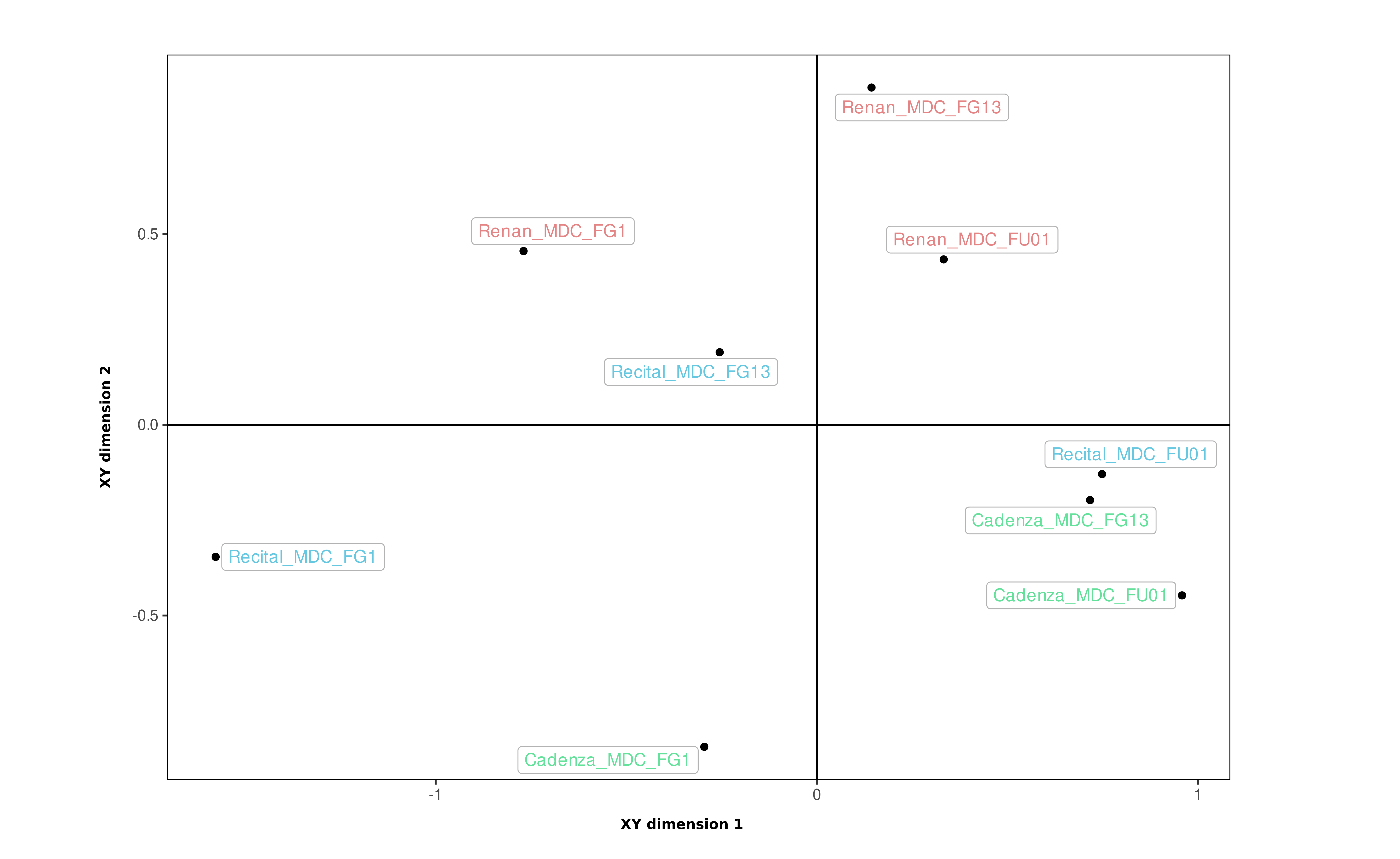

Supplement: Supplementary Figure 3 — rCCA unit representation plot from the two first components for all wheat cultivar – F. graminearum strain combinations. [file Image_3.TIFF]
